# Overviews on the cellular uptake mechanism of polysaccharide colloidal nanoparticles

**DOI:** 10.1111/jcmm.13110

**Published:** 2017-02-28

**Authors:** Sara Salatin, Ahmad Yari Khosroushahi

**Affiliations:** ^1^ Biotechnology Research Center Tabriz University of Medical Science Tabriz Iran; ^2^ Student Research Committee Tabriz University of Medical Science Tabriz Iran; ^3^ Department of Pharmaceutical Nanotechnology Faculty of Pharmacy Tabriz University of Medical Science Tabriz Iran; ^4^ Drug Applied Research Center Tabriz University of Medical Sciences Tabriz Iran; ^5^ Department of Pharmacognosy Faculty of Pharmacy Tabriz University of Medical Sciences Tabriz Iran

**Keywords:** drug, gene, endocytosis, nanoparticle, polysaccharide

## Abstract

Nanoparticulate drug/gene carriers have gained much attention in the past decades because of their versatile and tunable properties. However, efficacy of the therapeutic agents can be further enhanced using naturally occurring materials‐based nanoparticles. Polysaccharides are an emerging class of biopolymers; therefore, they are generally considered to be safe, non‐toxic, biocompatible and biodegradable. Considering that the target of nanoparticle‐based therapeutic strategies is localization of biomedical agents in subcellular compartments, a detailed understanding of the cellular mechanism involved in the uptake of polysaccharide‐based nanoparticles is essential for safe and efficient therapeutic applications. Uptake of the nanoparticles by the cellular systems occurs with a process known as endocytosis and is influenced by the physicochemical characteristics of nanoparticles such as size, shape and surface chemistry as well as the employed experimental conditions. In this study, we highlight the main endocytosis mechanisms responsible for the cellular uptake of polysaccharide nanoparticles containing drug/gene.

## Introduction

One significant obstacle for the development of effective drug/gene therapies, particularly anticancer therapies, has been their inability to cross the plasma membranes of cells. Plasma membrane provides a boundary between the cell and its environment to maintain the activities that are critical for the normal functioning of different types of cells 1. Biological membranes may block or limit specific accumulation of the therapeutic agents in an intracellular organelle of interest 2. On the other hand, the endosome or lysosome degradative compartment is not the final therapeutic goal; therefore, a search for effective strategies that are able to deliver therapeutic agents across the biological membranes and subsequently protect against the hostile hydrolytic environment of the lysosomes is necessary^3^. This is achieved using novel systems with high encapsulation capacity for targeted delivery of drugs, genes and diagnostic agents to specific cells or to particular intracellular components and release of their cargo in a sustained and time‐dependent profile 4.

Many polymeric nanoparticulate systems have been investigated to be appropriate for cell research and applications. Polymeric nanoparticles are ultrafine colloidal particles with the size range between 1–1000 nm and have different properties compared to their source materials, in which the therapeutic agents can be entrapped intra‐nanoparticles or can be incorporated *via* adsorption or chemical conjugation to the surface 5. Not only the synthetic polymers such as poly(lactide‐co‐glycolide) 6, polyacrylates 7, polycaprolactones 8 and polyethylenimine 9 but also natural polymers such as proteins 10, nucleic acids 11 and polysaccharides 12 have been used to prepare nanoparticulate drug/gene carriers. Over the years, it has been highlighted that nanoparticles frequently exhibit improved properties for easier translocation across biological barriers as they have the advantages of high surface area/volume ratio and increased mobility, and hence a great interactive potential with the biological surfaces 13, 14. Nanoparticles also offer the potential to protect the encapsulated molecules from both extracellular and intracellular degradation, resulting in improved intracellular bioavailability 15. In addition, surface conjugation of specific ligands allows nanoparticles to interact with a particular group of receptors at the cell and tissue surfaces, thereby favourably modifying the intracellular disposition of nanoparticles 16.

Following incorporation into the body, nanocarriers can translocate from their site of deposition to the elsewhere, such as the brain and bone marrow, by blood circulation system 17. The surface of nanoparticles is commonly coated with a layer of dissolved extracellular molecules in body fluids, such as proteins, sugars and lipids before their encounter with the cellular membranes, the so‐called protein corona 18. When reach the site of action, the loaded cargo may be released outside or cells take up nanoparticles and unload cargo at the desired intracellular compartment 19. Generally, the transport of macromolecular carriers such as nanoparticles from the cell surface to the lysosomal vesicles occurs with a process known as endocytosis 20. Following the nanoparticles uptake, they are able to significantly increase the drug concentration and act as an intracellular drug reservoir for long‐term release 4. Therefore, the use of natural polymer‐based nanoparticles seems to be advantageous as they are found virtually in all living organisms and their application does not exhibit toxic effects to the organisms owing to their intrinsic biocompatibility and biodegradability that ensure safe therapies 21.

Among natural polymers, proteins and polysaccharides tend to be associated with cells, rapidly internalized and degraded, thus enabling the intracellular release of the incorporated drug/gene from the transporter 22. As the use of polysaccharide‐based nanoparticles becomes more prevalent, it is necessary to address the interaction and uptake of this class of delivery vehicles by cellular systems 23. Therefore, in this review we discuss the recent progress on our understanding of the interaction between polysaccharide nanoparticles and cell membranes as well as the main endocytic pathway involved in their uptake.

## Mechanisms of nanoparticle endocytosis

The plasma membrane is a highly selective and effective barrier that protects all living cells from the surrounding environment and strongly limits the entry and exit of large macromolecular substances 24. Therefore, nanoparticulate systems need to overcome this barrier to intrude living cells 25.

It is well known that nanoparticulate systems are capable to enter live cells, often through the several endocytic pathways. However, passive penetration of the plasma membrane may occur as an alternative route. Upon endocytosis, nanomaterials are enclosed within the early endocytic vesicles and are thus not directly carried into the cytosol. In contrast, the nanomaterials internalized *via* membrane penetration are directly transferred into the cytoplasm, which can be the preferred choice particularly for the targeted drug delivery 26.

The term ‘endocytosis’ can be broadly divided into pinocytosis (cell drinking) and phagocytosis (cell eating). Pinocytosis is commonly involved in the internalization of fluids and molecules by small vesicles, and phagocytosis is the process by which the cells such as monocytes/macrophages, neutrophils and dendritic cells engulf large particulate matter and are able to form intracellular phagosomes 27. Pinocytosis can be further subdivided into four different basic categories namely macropinocytosis, clathrin‐mediated endocytosis, caveolin‐mediated endocytosis and clathrin‐ and caveolin‐independent endocytosis 18. These endocytosis mechanisms are found in specific types of cells and subsequently have a key role in the intracellular trafficking and fate of the captured particles 27. In addition, they are different in view of the coating composition, size of the detached vesicles and intracellular trafficking of the internalized material 28.

Macropinocytosis is a form of actin‐driven endocytic mechanism by which extracellular fluid and its debris are internalized in a non‐specific manner within large, heterogeneous vesicles, called macropinosomes 29. Each clathrin‐mediated internalization pathway is started by the specific interactions between ligands and extracellular receptors. Following entry into the cell, the internalized nanoparticles are typically trapped inside the endosomal/lysosomal vesicles, resulting in the degradation of the sequestered cargo material by the lysosomal enzymes 30. Caveolae/raft‐dependent endocytosis is involved in clustering of the lipid raft domains at the plasma membrane into the flask‐shaped invaginated structures called ‘caveolae’ and formed through the interaction of cellular membranes with the different types of proteins, especially caveolin 31. Endocytosis by clathrin‐coated pits or uncoated pits traffics the material to the lysosomal degradative compartment, while caveolae‐mediated endocytosis mediates the translocation to the Golgi apparatus, to endoplasmic reticulum or through the cell *via* trancytosis 32. Therefore, it is useful to develop other strategies such as caveolae***‐***mediated pathway and macropinocytosis which are somewhat non‐specific, and neither acidic nor digestive and can preferentially internalize nanoparticles through an alternative pathway, preventing lysosomal degradation after internalization 28.

**Figure 1:**
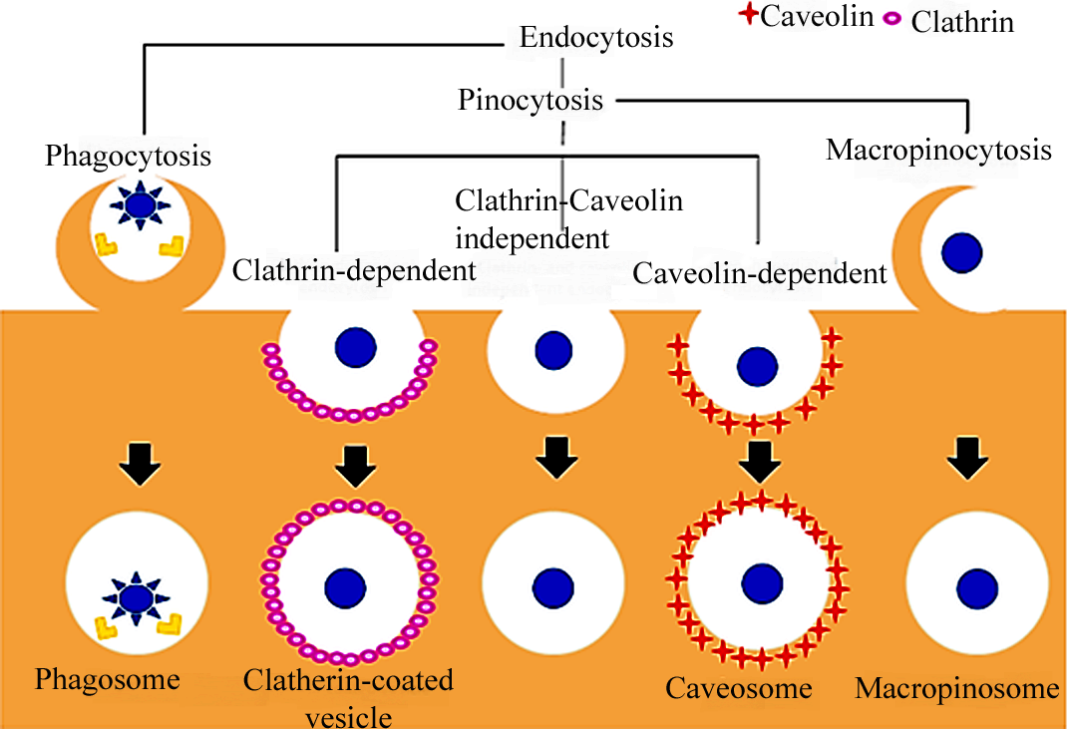
Scheme for the main pathways of nanoparticle endocytosis.

Notably, for *in vitro* quantitative assessment of cellular uptake, the nanoparticles could be labelled by fluorescent dyes or radioisotopes 33. On the other side, selective inhibition of the various endocytic process has been found to be a strong way for evaluating the cellular uptake of nanocarriers, although it is not complete in specificity 34. The main pathways ofnanoparticle endocytosis are illustrated in Figure 1.

## Factors affecting the efficacy of nanoparticle uptake

The efficiency of endocytosis depends on not only the size of nanoparticles, but also the charge, and surface coating 35. On the other side, culture medium and cell‐specific uptake properties could play a significant role in determining the interaction of nanoparticles with cell membranes 36. Optimization of the physicochemical parameters is required, in a specific model to each type of nanoparticles, to improve the efficiency of cellular uptake and intracellular drug delivery 28. In addition, the effects of various possible combinations of the nanoparticle characteristics must be evaluated to predict the nanotoxicity or prepare ideal nanoparticulate drug/gene carriers 37. Considering that each parameter can be changed, a large number of nanoparticle formulations could be designed.

### Size

The size of nanoparticles is of high importance for the evaluation of the performance and biological fate, particularly for addressing the efficacy of cellular internalization mechanism 38 as the size of nanoparticles is implicated in elimination by the mononuclear phagocytic system 39. The smaller the particle size, the easier they can be internalized by cells 14 as smaller particles have a larger surface area than same mass of larger particles, allowing them more contact with the biological membranes 40.

### Shape

**Figure 2:**
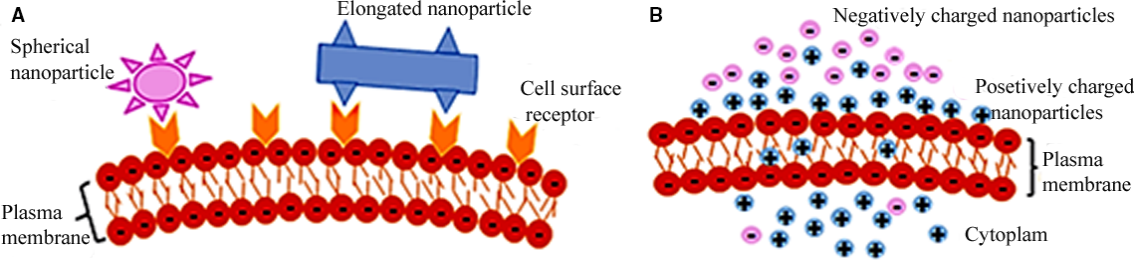
The effect of shape (**A**) and surface charge (**B**) on the nanoparticle–cell membrane interaction.

Likewise, shape has been found to dictate the biodistribution, blood residence time as well as the cellular uptake of nanoparticles 35. Elongated nanoparticles have been reported to yield a higher efficiency in adhering to the cells as compared to the spherical nanoparticles, especially after surface modification as elongated nanoparticles have a higher surface area and the ability to interact more efficiently with the cell surface membranes with respect to their shape 41. The interaction of elongated and spherical nanoparticles with cell membrane is presented in Figure 2A.

### Charge

The cell's plasma membrane interacts differently with positively or negatively surface***‐***charged nanoparticles. Generally, positively charged nanoparticles exhibit better internalization level (Fig. 2B) as a result of attractive electrostatic interaction with the negatively charged cell membrane that has a profound effect supporting the uptake of nanoparticles 41.

### Surface modification

Surface modification of the nanoparticles by cell‐specific targeting molecules has been reported as a powerful tool for increasing the cellular uptake 42. A wide range of ligands are available as a basis to improve targeted drug/gene delivery. The use of a specific ligand depends on several different parameters including the nature of target cell, the material used in the formulation of nanoparticles and the chemistry available for conjugating ligands to the nanoparticle surface 22.

Besides, intrinsic properties of some biomacromolecules, such as cell‐penetrating peptides (CPPs), for freely passing through the cell membranes in a non‐toxic manner suggests that they can be used for improving the intracellular availability of various cargoes such as small molecules, plasmid DNA (pDNA) and nanoparticles. Therefore, CPPs attached to the nanoparticles have been reported as an appealing strategy to enhance drug/gene delivery to and across biological barriers when compared with the non‐modified CPPs‐based nanoparticles 43. In addition, the coupling of CPP could offer the benefits of endosomal escape of its cargo 44.

### Experimental conditions

#### Cell type

Effective delivery of drug‐loaded nanoparticles into specific cells and subcellular targets under different conditions is of high importance in the field of biomedicine. For example, the main challenge in the treatment methods of cancer is to translocate pharmaceutical agents into cancer tumours without damaging healthy tissues 45. Cancer cells are different to non‐cancerous cells in various ways and divide at an unregulated pace, and most of the nanoparticles show different endocytic mechanisms in cancer and normal cells, offering a chance to design nanoparticles with high selectivity for targeted drug delivery 46.

#### Cell culture

Beyond the physicochemical characteristics of nanoparticles, the composition of common cellular culture media and the origin of serum mediate the nanoparticles uptake and cellular distribution 47. Nanoparticles do not behave as inert materials or soluble small molecules in solution phase. But rather, nanoparticles have been observed to undergo some aggregation/agglomeration processes and as a result they often show a tendency to form new classes of multisized molecular entities 48. Following entry into a biological fluid, the surface of nanoparticles is coated by a layer of biomolecules such as proteins and lipids that is usually referred to as bio‐corona and has been reported to have a key role in assessing the effective size, surface charge and aggregation state of the nanobiomaterials, and hence in determining of their biological behaviours such as biodistribution, complement activation, interaction with receptors at the cell surface and cellular uptake 41.

## Polysaccharide nanoparticles

**Figure 3:**
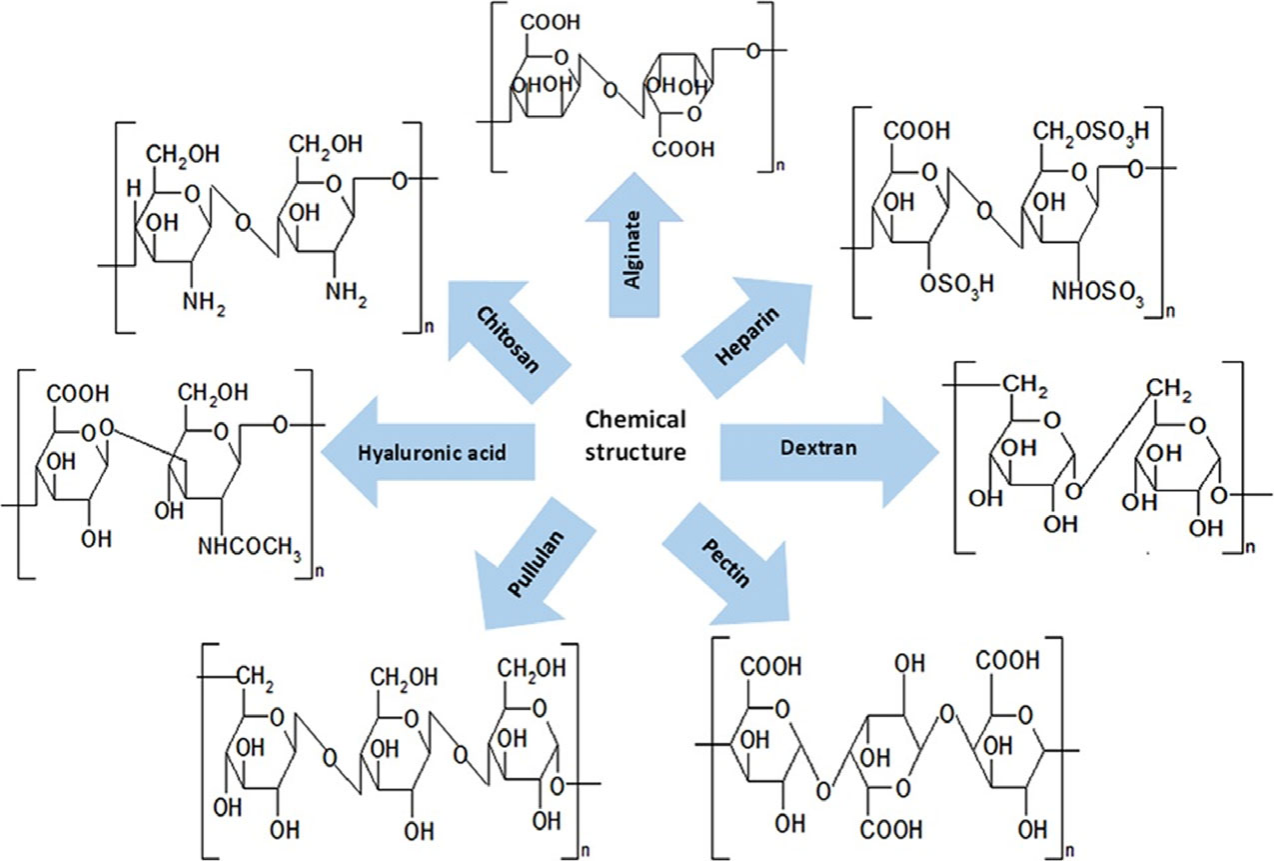
The chemical structure of polysaccharides.

Polysaccharides have been extensively used in pharmaceutics for development of drug/gene delivery systems. Polysaccharide materials can be classified in two groups including polyelectrolytes and non‐polyelectrolytes. Polyelectrolytes can be additionally divided based on their intrinsic charge including cationic (chitosan), anionic (alginate, heparin, pectin, hyaluronic acid) and neutral (pullulan, dextran) 49. The chemical structure of these polysaccharides has been shown in Figure 3.

Because of the amphiphilic nature, polysaccharide‐based materials can self‐assemble into ordered aggregates within aqueous environment and have various derivable groups on their molecular chains which can be easily adopted and modified with other chemical agents 21. Polysaccharides show a high affinity for mucosal surfaces covering the nasal, pulmonary and gastrointestinal tract. Besides, polysaccharides such as hyaluronic acid, pectin and heparin are capable of binding with cellular receptors at several levels 16. These properties of polysaccharides can be engineered to prepare desired type of nanoparticles with prolonged residence time at the target site, leading to enhanced permeation and bioavailability of the loaded biomolecules such as proteins and peptides in the absorption site.

### Alginate

**Figure 4:**
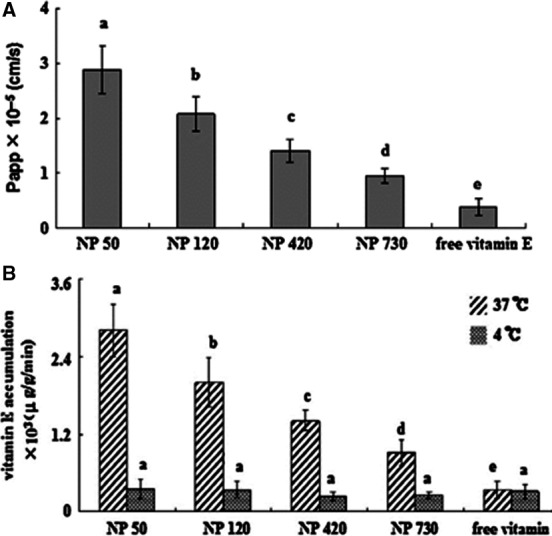
Permeability of vitamin E loaded in OAE nanoparticles across the Caco‐2 cell monolayers (**A**), accumulation of vitamin E loaded in OAE nanoparticles in jejunum at 37°C and 4°C (**B**) (with permission from authors and Carbohydrate Polymers)^38^.

Alginate is an anionic polysaccharide comprised of linear copolymers of guluronic and mannuronic acid residues and is now known to be a whole family of safe and haemocompatible polymers which do not show any significant accumulation within major organs and have provided evidence of *in vivo* degradation 50. In the presence of calcium ions, ionic interactions may occur between the glucuronic acid residues of alginate with the divalent calcium ions, leading to the gelation of alginate. Such property has allowed to the use of calcium alginate gel beads as one of the most frequently utilized materials in the pharmaceutical and medical fields 51. The effect of size and size distribution on the permeability and cellular internalization of the vitamin E‐loaded oleoyl alginate ester (OAE) nanoparticles has been demonstrated in an *in vitro* study (Caco‐2 cell line) and also an *ex vivo* study (excised rat jejunum). The authors reported that the loading capacity increased with increasing particle size, while transport of nanoparticles through Caco‐2 cells and also the cellular uptake in excised rat jejunum reduced (Fig. 4). Besides, results revealed that endocytic pathway of the OAE nanoparticles is size dependent and the main endocytic mechanisms are clathrin‐mediated endocytosis, caveolae‐mediated endocytosis and macropinocytosis 38. Similarly, kinetics and cellular uptake mechanism of nanoparticles formulated using dioctyl sodium sulfosuccinate (Aerosol OT™; AOT) and sodium alginate were evaluated *in vitro* using a model breast cancer cell line, for intracellular delivery of rhodamine. It was observed that incorporation of phospholipid into AOT–alginate nanoparticles can significantly enhance their uptake/intracellular accumulation, and metabolic inhibition studies suggested that cellular internalization of nanoparticles is mediated by endocytosis 52.

Folate–phytosterol–alginate nanoparticles have gained considerable attention as an ideal carrier of selective targeting drugs to cancer cells that overexpress the folate receptors, avoiding the possible cytotoxicity. Self‐assembled core/shell nanoparticles of phytosterol–alginate were prepared using the water‐soluble alginate substituted by hydrophobic phytosterols for delivery of doxorubicin (DOX). *In vitro* cellular tests showed that DOX‐loaded nanoparticles had a high binding affinity to the folate receptors and primarily located at the outer portion of the plasma membrane. It was observed that the nanoparticles are internalized by the folate receptor–mediated endocytosis mechanism, and this endocytosis pathway may result in a significant increase in the cellular uptake efficiency 53.

Alginates can electrostatically interact with the positively charged entities as a result of their negative charge, so they can be successfully used as an platform for delivery of cationic drugs and molecules 54. On the other side, formation of polyelectrolyte complex between the carboxyl groups of alginate and the amine groups of chitosan (CS) has been exploited to be suitable for the controlled release of drugs and other biological agents as compared to the alginate/CS formulation, alone 50. Folate‐conjugated CS–alginate complex nanoparticles were proven to efficiently mediate improved cellular uptake in HepG2 liver cells. The reason could be that folate was easily recognized by folate receptors on the surface of the HepG2 cells 55. Besides, the experiment showed that the transfection rate of pDNA into HEK293 cell could be increased when using alginate nanoparticles for gene delivery when compared to the CS/alginate‐ and CS nanoparticles, alone. Actually, not only alginate exhibits ‘proton sponge effect’, but also its degradation promotes the osmotic pressure, thereby increasing the endosomal release of cargo. Besides, swelling properties of the alginate owing to hydrogel property may mediate the cellular uptake and the release of pDNA into the cytosol 56.

### Chitosan

Chitosan (CS) is a naturally occurring linear polysaccharide composed primarily of repeating units of D‐glucosamine 17. It is structurally similar to the cellulose, and the presence of highly reactive amino groups renders the polymer a net positive charge 57. Chitosan is the most common polymer currently used for drug/gene delivery because of excellent properties such as good biocompatibility and natural antibacterial, anti‐inflammatory and neuroprotective behaviours, which ensure safe therapies 58. It can be enzymatically degraded *in vivo* by enzymes such as lysozyme and chitosanase, into oligomers and finally to *N*‐glucosamine, which is endogenously present in the human body 59.

The most common method to prepare CS nanoparticles is ionic gelation. This method offers a simple and rapid preparation technique in the aqueous environment and is based on the electrostatic interaction of the amine groups of CS and the negatively charged groups of a polyanion such as tripolyphosphate (TPP). The size and surface characteristics of particles can be easily adjusted by varying the ratio between the polymer and polyanion 60.

It has been shown that CS nanoparticles with positive charge tend to make interactions with the negatively charged proteins at the cell surface to form clathrin vesicles. Whenever vesicles fall off from the plasma membrane into the cytoplasm, they can fuse with endosomes/lysosomes compartments. Then, acidity of the environment enhances the breaking of chemical bonds and structure of particle by enzymes, leading to the rapid release of cargo. *In vitro* data demonstrated that CS nanoparticles with positive surface and good spherical monodispersity enhanced the cellular uptake of the molecular cargos, including the anti‐cancer drug gefitinib and the lysosomal targeting drug chloroquine through the caveolae‐mediated endocytosis and macropinocytosis pathway 61.

Comparing the cellular uptake of CS molecules and nanoparticles and hence their capability to mediate insulin passage through Caco‐2 cell monolayers showed two times higher interaction with nanoparticles than that of CS molecules upon 2‐hrs incubation at the loading concentration of 1 mg/ml. Using chlorpromazine to reduce the number of coated pit‐associated receptors on the cell surface by disrupting the regulation of clathrin assembly/disassembly, it was concluded that clathrin is the major route for cellular uptake of the CS nanoparticles. These findings suggest that the structural transformation of CS molecules arranged in microscale linear chains into the condensed nanoscale particle results in a greatly improved cellular interaction and transport across the Caco‐2 cell monolayers 62. In another study, uptake of CS nanoparticles into Caco‐2 cells was reported to take place through a similar pattern to that of CS molecules, and the uptake of approximately 95.1% of CS nanoparticles and 86.0% of CS molecules was mediated through an energy‐dependent endocytosis. It was indicated that CS nanoparticles can cause stronger changes in the distribution of membrane proteins, fluidity of membrane lipids and general membrane composition 63. Besides, mechanism of uptake of CS nanoparticles, in contrast to CS molecules, exhibits saturable kinetics and their uptake capacity decreases with decreasing molecular weight and also degree of deacetylation 59.

The effectiveness of CS at absorption site is limited because of poor water solubility at pH > 9. As the pH increases above 6, CS starts to lose its positive charge density, induces rapid formation of aggregates and precipitates. To solve the issue above, thiolated CS (TCS) have been developed as a new generation of bioadhesive polymers 64. TCS binds to the mucosal surfaces with a higher avidity, which is based on the disulphide bond formation between thiol groups of the polymer and cysteine‐rich subdomains of glycoproteins in the mucus. These covalent bonds are very stronger than non‐covalent bonds, ultimately leading to an increase in cellular uptake 65. The experiment reported that the degree of uptake of TCS–sodium alginate (SA) nanoparticles into the human corneal epithelium was importantly higher than that of CS–SA nanoparticles. This may be of more emphasis on the fact that TCS–SA nanoparticles are more mucoadhesive and so more efficient as a drug carrier for ocular delivery as compared to the CS–SA nanoparticles 64.

**Figure 5:**
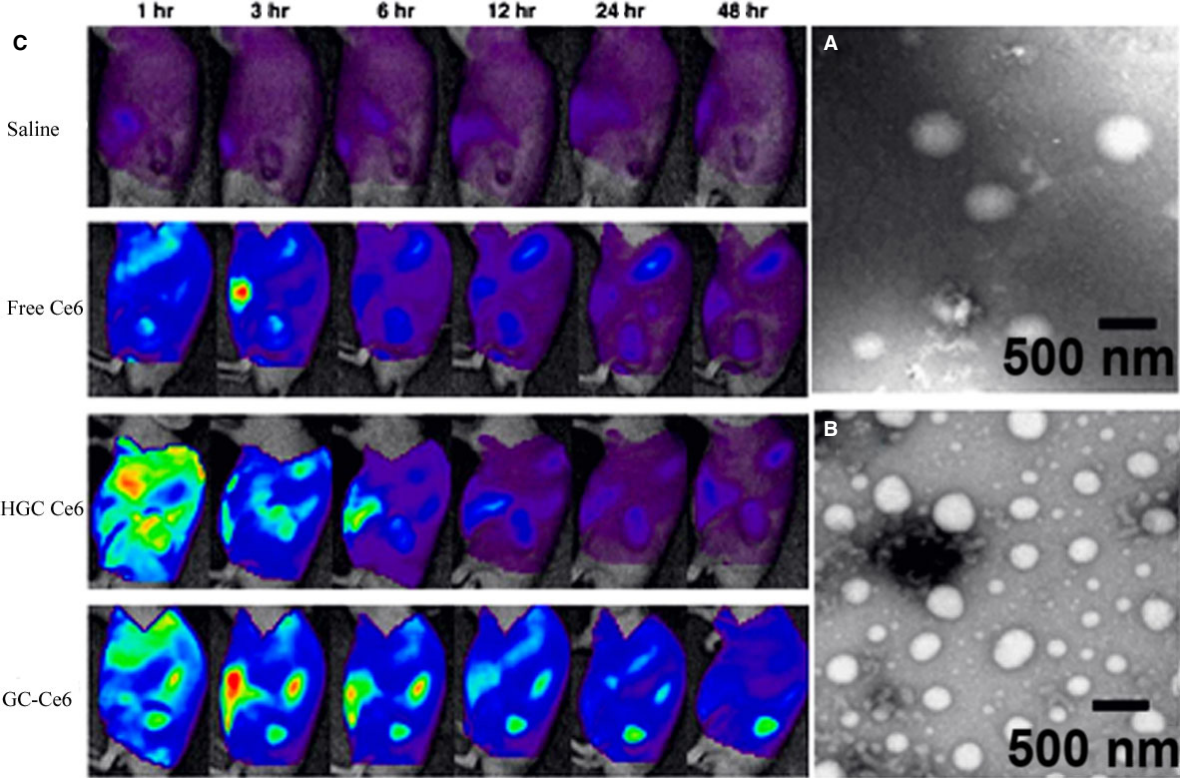
TEM images of HGC‐Ce6 (**A**) and GC‐Ce6 nanoparticles (**B**). *In vivo* time‐dependant whole‐body imaging of athymic nude mice bearing HT‐29 tumours after i.v. injection of saline, free Ce6, HGC‐Ce6 or GC‐Ce6 (2.5 mg/kg of Ce6) (**C**) (with permission from authors and Journal of Controlled Release) 67.

Glycol CS is commercially available as another derivative of CS with enhanced water solubility and has been reported in a large scope in the field of diagnostic imaging and drug delivery 66. To evaluate the uptake mechanism of hydrophobically modified glycol CS (HGCS) nanoparticles, HeLa H2B‐GFP cells were preincubated with the selective inhibitors of caveolae formation, clathrin association and Na^+^/H^+^ exchange to block the specific endocytic pathways. The results showed that HGCS nanoparticles do not forcefully follow one of the main endocytic pathways and the majority of them were taken up through the macropinocytosis which is a non‐destructive route as compared to the clathrin‐mediated endocytosis. Notably, HGCS nanoparticles exhibited different internalization efficiencies and also intracellular behaviours depending on their uptake route and were able to impair the lysosomal degradation process *via* fusion 19. In an alternative study, HGCS nanoparticles had a globular shape with the mean diameter of 359 nm and exhibited an increased distribution in the whole cells through several non‐destructive endocytic pathways when compared to the parent HGCS polymers 32. HGCS nanoparticles with the physically entrapped chlorin e6 (HGC‐Ce6) as well as the chemically entrapped chlorin e6***–***conjugated GCS nanoparticles (GC‐Ce6) were synthesized as two kinds of tumour‐targeting nanoparticles containing photosensitizers for photodynamic therapy. Both nanoparticles had a spherical morphology (Fig. 5A and B) and a small particle size (300–350 nm), but HGC‐Ce6 showed a burst of drug release in the buffer condition as compared to the GC‐Ce6. Tumour cells incubated with HGC‐Ce6 or GC‐Ce6 nanoparticles exhibited efficient cellular uptake of both nanoparticles. However, in the case of cells treated with GC‐Ce6, fluorescent spots of Ce6s were more than those HGC‐Ce6, demonstrating that Ce6 of GC‐Ce6 was chemically conjugated to the GC polymers and could not be released from the nanoparticles. When injected into HT‐29 tumour‐bearing mice through the tail vein**,** HGC‐Ce6 did not accumulate efficiently in tumour tissue, as a result of rapid release of the physically loaded drug, while GC‐Ce6 exhibited a more prolonged circulation profile and higher tumour accumulation, resulting in high therapeutic efficacy in cancer treatment (Fig. 5C) 67. Similarly, nanoparticles prepared from *N*,*O*‐carboxymethyl CS, a water‐soluble derivative of CS, were more efficient in promoting cellular uptake and apoptosis in breast cancer cell lines against normal cells 68.

CS has also been explored as a means to enhance mucoadhesive and absorption‐enhancing characteristics. This property is based on the interaction between the positively charged amino groups of CS and negatively charged residues in the mucin 69. A different study, by Behrens *et al*., compared the interaction of hydrophobic polystyrene‐, mucoadhesive CS‐ and stealth‐like PLA–PEG nanoparticles with cellular membranes using two intestinal cell culture models: Caco‐2 as a suitable model for enterocytes in the small intestine which are lack in the mucus layer and also MTX‐E12 cells as mucus‐secreting goblet cells. The results demonstrated that the cellular interaction of nanoparticles was significantly different depending on their physicochemical features and type of the cell line. The following rank order was obtained in Caco‐2 cell line: Polystyrene > CS ≫ PLA–PEG and in MTX‐E12 cells: CS > polystyrene ≫ PLA–PEG. In this case, the mucus layer covering enterocytes and goblet cells is a major barrier for nanoparticle absorption and CS nanoparticles displayed a stronger association and internalization with mucus‐secreting cells MTX‐E12 than with Caco‐2 cell monolayers as a result of its electrostatic interactions 70. In addition, chuah *et al*. 14 reported that the mucoadhesive interaction and uptake of the model molecule coumarin into the colorectal cancer cells could be increased when using CS‐based nanoparticles.

CS has been extensively studied as a vehicle for gene transfer into a wide range of cells because of its high efficiency to condense DNA and siRNA 71. Positively charged CS can compact negatively charged nucleic acids in the form of nanoscale complexes, resulting in protection of the nucleic acids from degradation and improved cellular uptake as compared to the naked nucleic acids 34. After self‐assembly, the highly negative charged nature of nucleic acids becomes neutralized rapidly, and the surface charge of the obtained polyplex gets positive at higher *N*/*P* ratio. In fact, this positive charge is required for effective binding to the anionic cell surface, and consequently leading to greater cellular uptake 71. In addition to electrostatic complexation with the negatively charged nucleic acids, CS alone is able to promote endosomal escape of the loaded cargos into the cytosol by ‘proton sponge’ effect 72. An attempt was made to optimize the transfection efficiency of CS in HeLa cells by a new generation of self‐branched trisaccharide‐substituted CS oligomers (SBTCO). The uptake of linear CS (LCO) was approximately 10 times lower than that of the SBTCO polyplexes. It is because of this that the SBTCO polyplexes are taken up by a clathrin‐independent endocytic mechanism and escape from endosomal vesicles, successfully. On the other hand, LCO polyplexes, which aggregate into bigger nanoparticles and associate strongly to the cell surface, are internalized to a less amount through different endocytic pathways and are unable to escape to the cytosol 34.

However, it is important to note that strong electrostatic interaction between CS and nucleic acid prevents dissociation of the nucleic acids within cells, thus precluding the nucleic acids transcription and resulting in poor transfection efficiency 73. The strength of this can be reduced in the presence of a secondary anionic polymer 74. Application of various anionic synthetic polymers such as Poly(l‐lysine) 75 or Polyethylenimine 76 have been previously reported. In this context, alginate can be used as a non‐toxic and biocompatible secondary polymer in combination with CS. Further supporting this hypothesis, we observed that CS/alginate nanoparticles mediated cellular uptake of antisense better than naked antisense in T47D cells 74. CS and CS/alginate nanoparticles showed better capacity to load pDNA and had the higher transfection efficacy into HEK293 cells than alginate nanoparticles 56.

The surface modification of CS nanoparticles by ligands having high affinity to the specific cell surface receptors such as lactose, transferrin, folate and mannose promotes their cell specificity and binding/transfection efficiency through receptor‐mediated endocytosis 77. For example, mannose receptor which is a transmembrane glycoprotein abundantly expressed on the surface of antigen‐presenting cells could be recognized by mannose residues of molecules 78. Observations have confirmed that the ability to bind specifically to macrophages and transfection efficiency of mannosylated CS nanoparticles containing gastrin‐releasing (pGRP) peptide were significantly higher than those of uncoated particles 77 as macrophages (such as Kupffer cells) express a range of mannose specific membrane receptors which uptake glycoproteins bearing high mannose chains by clathrin‐coated vesicles for delivery into the endosomal system 79. Furthermore, mannosylated CS nanoparticles exhibited a much higher transfection efficiency into Raw 264.7 cells (bears mannose receptors) than HeLa cells and successfully found as an ideal targeting gene delivery vehicle to the macrophages 71. Oleoyl CS nanoparticles have been employed as another promising system for the delivery of hydrophobic antitumour agents. *In vitro* studies demonstrated that the cellular uptake of oleoyl CS nanoparticles was probably caused by adsorptive endocytosis, which is preceded by non‐specific interaction of ligand with the cell membrane 4. Another research focus has been to study the cellular uptake of both trimethyl CS nanoparticles and their goblet cell‐targeting CSK (CSKSSDYQC) peptide–modified nanoparticles in Caco‐2/HT29‐MTX‐cocultured cells as well as in Caco‐2 cells. It was reported that both treated and untreated nanoparticles can deliver a remarkably higher quantity of FITC‐insulin through caveolae‐mediated endocytosis and macropinocytosis routes as compared to free FITC‐insulin solution in both cell models. However, the amount of uptake of CSK‐modified nanoparticles in cocultured cells was 2.21‐times higher than that of un‐modified nanoparticles, while there was no significant alteration between the two nanoparticles in Caco‐2 cell. These data suggested that the improved uptake of modified nanoparticles was mediated *via* specific interaction between the CSK peptide and the mucus‐producing goblet cell‐like HT29‐MTX cells 80. To improve the ability of CS nanoparticles to efficiently transform hepatic cells, galactosylated CS nanoparticles were prepared. Surface modification of CS nanoparticles demonstrated that they have a significantly enhanced efficiency in the intracellular delivery of silymarin for the treatment of liver cirrhosis by hepatic asialoglycoprotein receptor‐mediated binding 81. Consequently, alginate‐coated CS nanoparticles have been used to allow crossing of the membrane barriers as well as being taken up into the rat Peyer's patches 82.

Besides, conjugation of CS with CPP is expected to drastically promote the cellular uptake of nanoparticles as a result of the intrinsic features of CPP to translocate across the plasma membrane and, therefore, could be used for delivery of bioactive molecules into different types of cells 83. For example, the CS nanoparticles attached with trans‐activating transcriptional activator (TAT) protein of HIV‐1 exhibited significant increased cellular accumulation when compared with un‐modified nanoparticles 84. A similar study was also employed to the Tat‐related peptide so that nanoparticles prepared by combination of CS–HIV‐1 Tat peptide and CS–thioglycolic acid conjugates showed an enhanced transfection efficiency of pDNA in HEK293 cells, 7.12‐ and 67.37‐times greater than that of the unmodified CS and pDNA alone 85.

### Heparin

Heparin is a water‐soluble anionic and extremely sulphated polysaccharide coupled with a variety of biological activities such as anticoagulation, anti‐inflammatory and antiangiogenesis effects 86. In addition, the antitumour effects of heparin and its derivative, low molecular weight heparin (LMWH), has been reported by several authors 87, 88, 89. The antitumour activity of heparin may be related to its ability to suppress the tumour angiogenesis and metastasis processes *via* disruption in the activity of VEGF and bFGF 90. Therefore, the use of chemical and biological activities of heparin to develop more efficient nanoparticulate delivery systems opens the door for novel safe therapeutic applications.

Heparin has been described to design self‐assembled micelle‐like nanoparticles after its conjugation with hydrophobic polymer or moieties, and the size of micellar structures can be precisely optimized using a controlled coupling ratio of that hydrophobic moiety. Heparin nanoparticles have a large number of reactive functional groups and can be engineered to provide a large ratio of surface area for introducing various types of tumour‐targeting ligands, optical imaging agents and biomolecules 91. On the other hand, some protein components such as different growth factors contain the heparin‐binding domains that mediate their interaction with heparin‐based nanoparticle surfaces 92. Therefore, heparin nanoparticles can serve as a useful system to protect the heparin‐binding growth factors against the proteolytic and chemical degradation and enhance the interaction between the growth factors and their receptors 93.

Self‐assembled nanoparticles were prepared using LMWH–stearylamine (SA) conjugates (LHSA) for cell uptake of anticancer drug, docetaxel, wherein the LMWH was the hydrophilic segment and SA as its hydrophobic counterpart. A hydrophobic minor core could facilitate interaction with the plasma membrane and enhance cellular uptake efficiency. As a result, the LHSA nanoparticles loaded with coumarin 6 exhibited significantly increased accumulation in 6 MCF‐7 and MDA‐MB‐231 human breast cancer cells when compared with the coumarin 6 solution 94.

### Hyaluronic acid

Hyaluronic acid (HA), also called hyaluronan, is one of the main constituent components of the extracellular matrix, present abundantly in the various organs including connective, epithelial and neural tissues 95. It is a biocompatible and biodegradable linear polysaccharide with good chemical stability and considerable water‐absorbing capabilities 96. The carboxylic acid groups in the structure of HA provide one of the best functional moieties to be conjugated to an optimal number of drugs or targeting ligands 97.

Core–shell polymeric structures can be prepared by conjugation of hydrophobic moieties such as tetradecylamine or bile acids with the carboxylic acid groups of HA through carbodiimide chemistry, thereby encapsulating of therapeutic and/or imaging molecules into inner hydrophobic cores 98. These outstanding properties allow HA potential applications in pharmaceutical and medical sciences.

**Figure 6:**
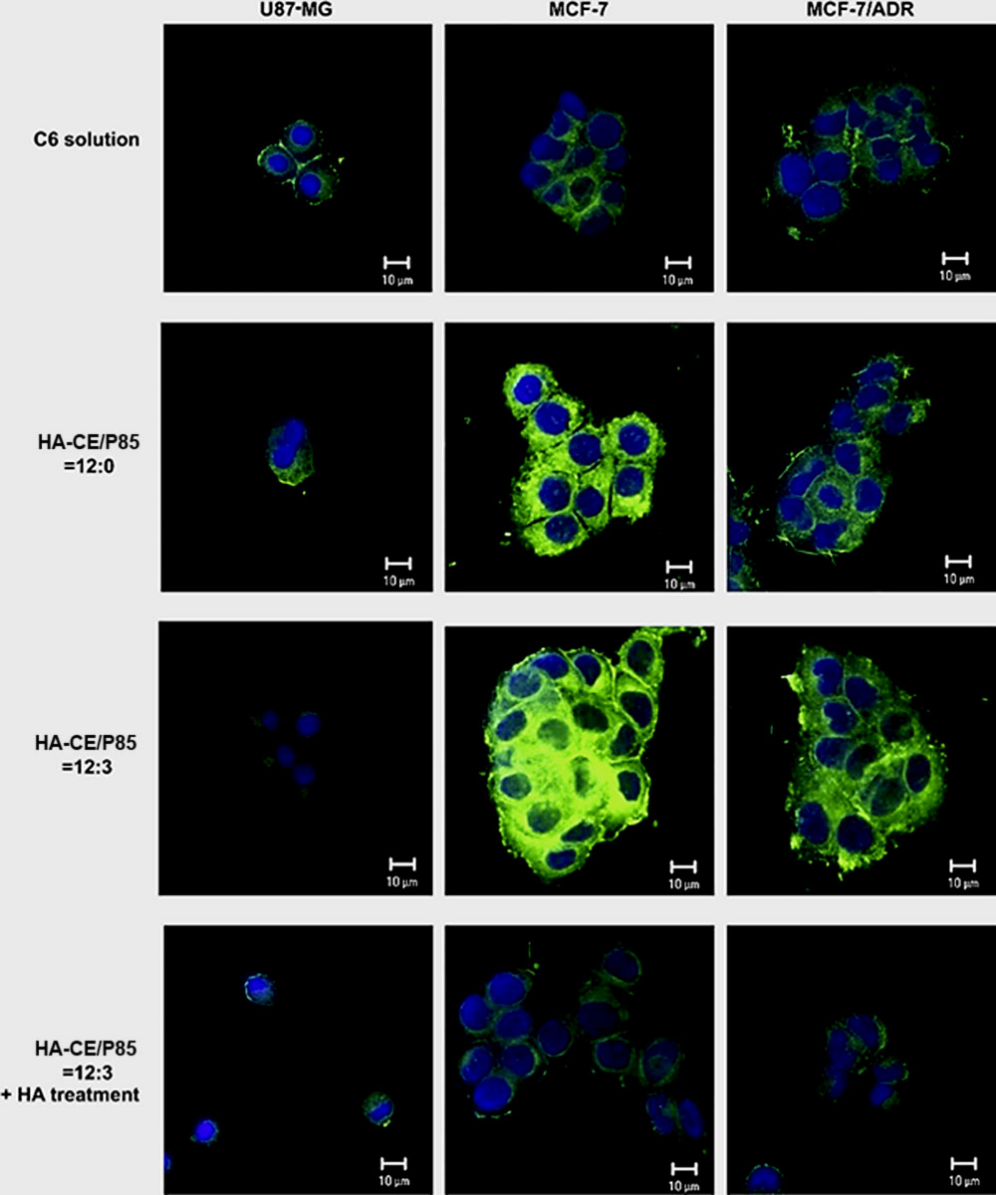
*In vitro* cellular uptake studies of coumarin 6‐loaded nanoparticles in U87MG, MCF‐7 and MCF‐7/ADR cells observed by CLSM. Green and blue colours indicate coumarin 6 and DAPI, respectively (with permission from authors and Biomaterials) 95.

**Figure 7:**
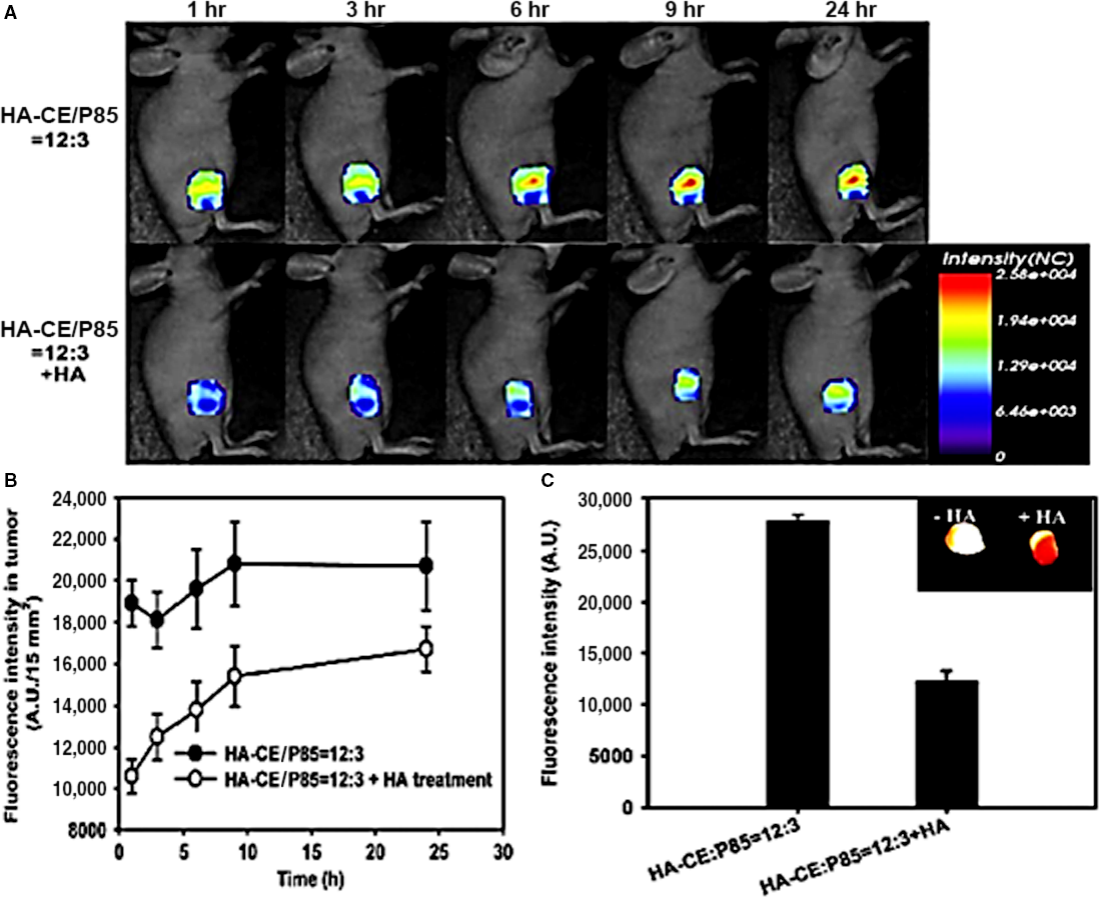
Fluorescence imaging of DCT‐loaded HA‐CE/P85‐based nanoparticle in a tumour‐bearing mouse. Time‐dependent *in vivo* images of mouse bearing MCF‐7/ADR tumours after intravenous injection of nanoparticles (**A**). *In vivo* fluorescence intensity profiles according to time in tumour tissues (**B**). *Ex vivo* fluorescence intensities of dissected tumours one day after the injection of nanoparticles. Inset indicates *ex vivo* fluorescence image. Error bar means S.D. (*n* ¼ 3) (**C**) (with permission from authors and Biomaterials) 95.

It was reported that HA nanoparticles are capable of crossing Caco‐2 cell monolayers and increasing the cellular uptake of the insulin as compared to the insulin solution 99. Notably, HA shows high binding affinity towards the CD44 and hyaluronan‐mediated motility **(** *RHAMM* **)** receptors overexpressed by a number of tumour cells and hence the high passive targeting ability towards tumour cells 100, 101. Therefore, HAs are taken up into the cells by receptor‐mediated endocytosis and then are transported to the lysosome, where they are ultimately degraded by lysosomal enzyme, Hyal‐1^102^. HA‐based nanoparticles are considered to be an effective system to easily deliver anticancer drugs into the CD44‐overexpressing tumour cells through the receptor‐mediated endocytosis, and the subsequent enzymatic degradation results in the sustained release of the nanoparticle's content 103, 104. In a primary study, Hua *et al*. 105 demonstrated experimental evidence for binding and uptake of HA by chondrocytes through the CD44*‐*mediated endocytosis mechanism. Similarly, authors exhibited experimental data for binding and uptake of the near‐infrared Cy5.5–labelled 5β‐cholanic acid–conjugated HA (HACA) nanoparticles by liver sinusoidal endothelial cells expressing HA receptors as well as phagocytic cells of the reticuloendothelial system. Meanwhile, this study compared the tumour targetability of PEGylated‐ and non‐PEGylated HA nanoparticles as application of HA nanoparticles for tumour therapy and diagnosis is limited because of their preferential accumulation in the liver site after systemic administration. The experiment revealed that PEGylated HA nanoparticles resulted in a 1.6‐fold higher accumulation in the cancer cells (SCC7, MDA‐MB‐231 and HCT116) when compared to the bare HA nanoparticles 100. Another study namely by Huang *et al*. 106 showed that accumulation level of polyelectrolyte nanocomplexes designed by HA‐grafted polycaprolactone (HA‐g‐PCL) nanoparticles in cancer cells (EC109) was significantly greater than by normal fibroblasts (NIH3T3). Nanoparticles composed of various concentrations of HA, ceramide and Pluronic 85 (HA‐CE/P85) were formulated for intravenous docetaxel (DCT) delivery. P85 is referred to as an efficient block copolymer that likely interacts with biological cell membranes upon increasing the length of the hydrophobic block from 30 to 60. Intracellular uptake efficiency was evaluated by Cy5.5–labelled HA‐CE–based nanoparticles in the three cell lines including U87MG (low CD44 expression), MCF‐7 (high CD44 expression) and MCF‐7/ADR (high CD44 expression and showing MDR to anti‐cancer agents). The fluorescence signals from the HA‐CE/P85 = 12:0 and HA‐CE/P85 = 12:3 groups in MCF‐7 cells were higher than those of U87MG cells. However, blocking of the CD44 receptor by HA treatment decreased cellular uptake in MCF‐7 cells (Fig. 6). The *in vivo* tumour targetability of nanoparticles was also demonstrated in MCF‐7/tumour‐bearing mice by non‐invasive near‐infrared fluorescence imaging (Fig. 7) 95.

HA has also been used to enhance the transfection efficiency of CS by facilitating interaction with cell surface receptors, such as CD44 107.

**Figure 8:**
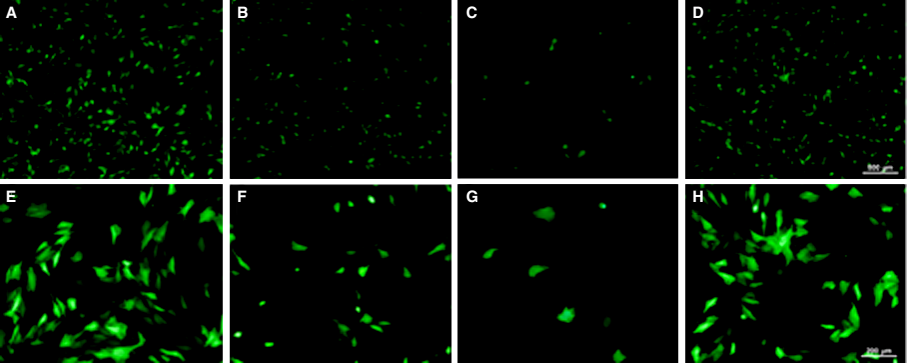
Images of chondrocytes transfected with HA/CS–plasmid nanoparticles, CS–plasmid nanoparticles, naked plasmid DNA and LipofectamineTM 2000 as observed under fluorescence microscope (100× magnification for (**A**)–(**D**); 200× magnification for (**E**)–(**H**)). HA/CS–plasmid nanoparticles (**A** and **E**); CS–plasmid nanoparticles (**B** and **F**); naked DNA (**C** and **G**); and LipofectamineTM 2000 (**D** and **H**) (with permission from authors and International Journal of Pharmaceutics) 95.

A new non‐viral gene vector to primary chondrocytes consisting of hybrid HA/CS nanoparticles was described by Hua *et al*. In this study, HA/CS nanoparticles were found to be far more effective in transporting biologically active pDNA into cells and fluorescence intensity of EGFP also increased significantly than that of CS nanoparticles under the same conditions, as shown in Figure 8 108. A similar strategy was described for bioadhesive HA/CS nanoparticle, which was found to form stable complexes with pEGFP or pβ‐gal, and to promote its cellular uptake in different cell lines 109. Besides, it was observed that pDNA‐loaded HA/CS nanoparticles can enter into both corneal and conjunctival cells and are located at the cellular periphery by hyaluronan receptors through a caveolin‐dependent endocytic mechanism 110. In 2013, So *et al*. demonstrated that nanoparticles containing HA and TCS could be used as a powerful tool for gene delivery to spinal cord. It was shown that HA/CS nanoparticles are not cytotoxic and able to efficiently deliver pDNA into neuronal cell as a result of hyaluronan receptor mediation 111.

### Dextran

Dextran is an unbranched water‐soluble polysaccharide and has been used in a range of biomedical applications because of its excellent biocompatibility, biodegradability, non‐antigenicity and non‐immunogenicity properties 112. Hydroxyl groups at the dextran chain allow further chemical conjugation with targeting ligands and biomedical agents (*i.e*., proteins, aptamers or pharmaceutical agents), and its disintegration occurs mainly by dextranase enzyme which is found in liver, spleen, kidney and bottom section of the gastrointestinal tract 113. Besides, dextran can be considered as an interesting alternative choice to PEG hydrophilic segments when designing the amphiphilic block copolymers 114. Therefore, dextran has now been the subject of numerous studies to design the stealth nanoparticles as a result of the escape from RES and as it enhances blood circulation time 115.

Mannosylated dextran nanoparticles have been shown to involve enhanced cellular association as well as higher antigen delivery to dendritic cells (DCs) when compared to unmodified particles or particles modified with a non‐specific sugar residue for design and development of optimally modulated vaccine delivery system 116. In addition, Yu *et al*. 117 demonstrated that cross‐linked nanoparticles generated by dextran–lipoic acid derivatives were able to cross cell membranes and to translocate DOX into the nuclei of cancer cells (HeLa and RAW264.7) as compared to the free DOX.

Dextran–spermine is a biodegradable and water‐soluble cationic polymer which is prepared by reductive amination synthesis between oxidized dextran and the naturally occurring tetramine spermine. Dextran–spermine complexes have been found to be active in transfecting a wide range of cell lines *in vitro* through their electrostatic interactions with cell membranes 118. It was reported that positively charged dextran–spermine nanoparticles were able to interact with the anionic substances on the cell surface and can be used for gene delivery 119. These results were in agreement with other study performed in COS‐7 cell line, which revealed that dextran–spermine nanoparticles containing pDNA are internalized mainly by electrostatic interaction 120. Another experiment showed that mixing PEG with cationic dextran–spermine nanoparticles resulted in a new class of non‐viral vectors that significantly improve transfection efficiency with a marked reduction in the amount of cytotoxicity in the leukaemic cells. Here, PEG seems to be very efficient to mediate the protection of pDNA/dextran–spermine complexes from interaction with enzymatic degradation and plasma proteins 114.

### Pullulan

Pullulan is a highly water‐soluble linear neutral polysaccharide produced from the fermentation activity of strains of fungus *Aureobasidium pullulans* 121. Several advantages have been experimentally reported about pullulans that make them suitable for drug delivery applications such as ability to adhere and form fibres as well as thin biodegradable films. Adhesion of pullulan‐based carriers to cell surfaces may be advantageous in drug/gene delivery applications with increasing the retention time and consequently allowing for timely absorption of the entrapped cargos 122. In addition, pullulan exhibits high affinity for asialoglycoprotein receptors overexpressed on hepatocyte cells and hence can be specifically internalized by hepatocytes through the receptor‐mediated endocytosis 123. A comparative study, by Li *et al.,* performed recently to assess intracellular distribution of pullulan–DOX conjugate nanoparticles in HeLa, HepG2 and L929 cells. It was observed that uptake of nanoparticles by HepG2 cells was significantly higher than HeLa and L929 cells. These results provide evidence that pullulan residues of nanoparticle shell act as targeting moieties and are able to mediate uptake of nanoparticles by receptor‐mediated endocytosis through ASGPR receptor recognition on HepG2 cells. The ASGPR is not highly expressed at the surface of HeLa and L929 cells 42. A similar experiment was also applied to the folic acid–decorated maleilated pullulan–DOX (FA–MP–DOX) conjugates. When FA–MP was conjugated to the DOX, it showed an increased efficiency of drug delivery in ovarian cancer cell line A2780 which was an FA receptor expressing cancer cell line. The possible mechanism is that DOX conjugates would transport many DOX molecules, when they were taken up by cells through receptor‐mediated endocytosis, resulting in the higher concentration of DOX than that of passive diffusion 124.

Carboxymethyl pullulan (CMP) was reported as a polyanion for the first time to prepare composite nanoparticles for delivery of vaccines 125. The process of carboxymethylation enhances solubility of pullulan in cold water, and adding carboxylate groups along the polymer backbone provides a negative charge which is then allowed to react with polycations to form stable nanocomplexes during polyion complexation 126. In contrast to other organs, CMPs exhibit higher binding affinity to the spleen and lymph nodes 127. Nanoparticles made by electrostatic interaction between negatively charged CMP and positively charged CS derivatives (*N*‐trimethyl CS chloride, CS glutamate, CS chloride) showed a significantly low cytotoxic activity in respiratory cells (Calu‐3 cells) than that of the CS derivatives, alone. Much stronger complexation between *N*‐trimethyl CS chloride and CMP resulted in smaller particle size when compared with CS salts. In mouse macrophages (J774A.1), the highest uptake efficiency was found with *N*‐trimethyl CS chloride–CMP composite particles after 2‐hrs incubation which could be explained by their smaller dimension and more narrow particle size distribution 125.

**Figure 9:**
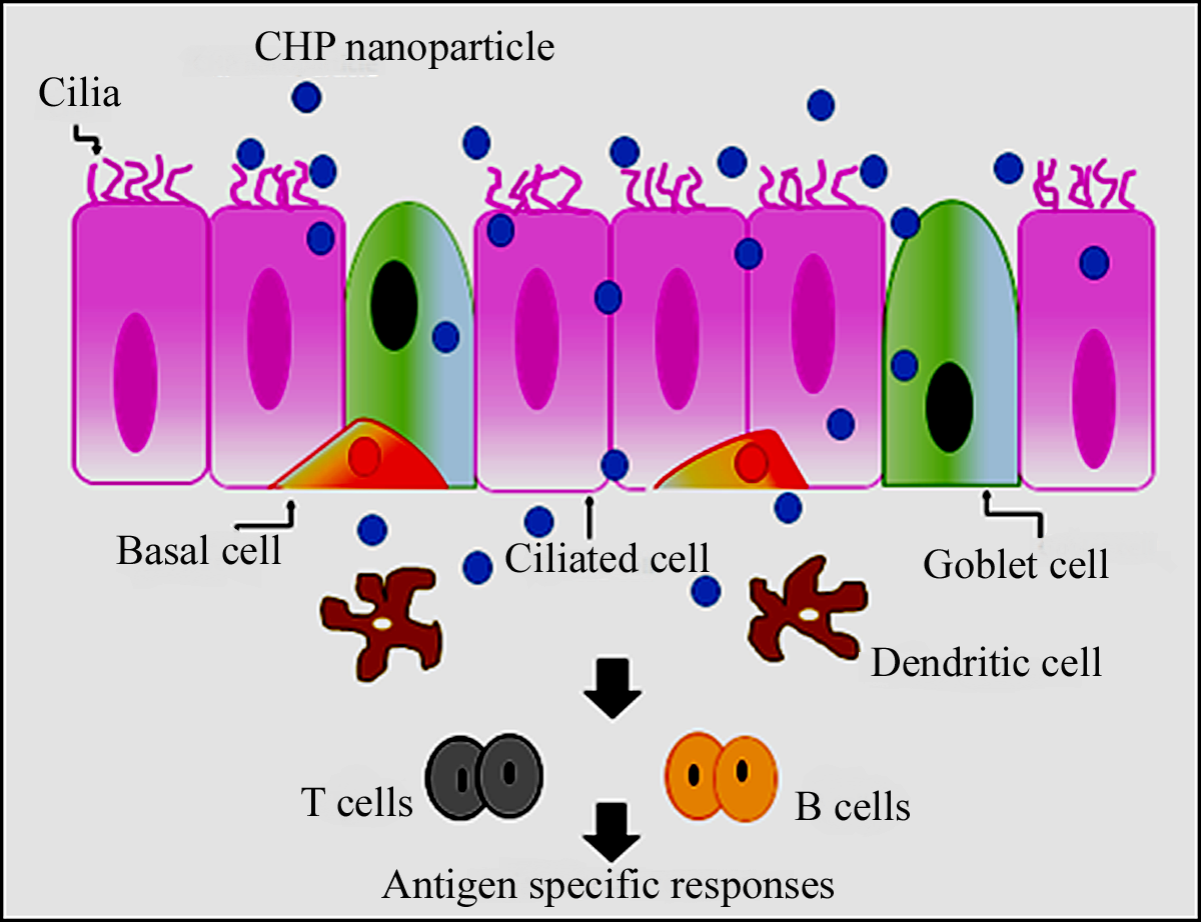
Schematic representation of the intranasal vaccine delivery by CHP nanoparticles.

Cholesteryl pullulan (CHP) is the naturally occurring polysaccharide, pullulan*,* which chemically modified with hydrophobic cholesteryl moieties 128. In aqueous solution, CHPs gather together to form self‐assembled nanoparticles and their interior cavity allows for the encapsulation of pharmaceutical payloads through the hydrophobic interactions 129. It has been found that CHP nanoparticles are effectively transferred to antigen‐presenting cells such as DCs and/or macrophages, allowing for a stronger immune response 130. Besides, CHP nanoparticles have been widely exploited in the nasal vaccine delivery because of their ability to adhere to the nasal epithelium 125. Figure 9 shows the uptake of vaccine antigen‐loaded CHP nanoparticles by nasal dendritic cells as a result of the induction of antigen specific responses. Daiki *et al*. reported on the immune‐enhancing ability of the tumour necrosis factor‐α–encapsulated CHP nanoparticles to act as a vaccine adjuvant for inducing systemic IgG1 as well as mucosal IgA *via* the nasal route of administration. As a result, these nanoparticles promoted antigen uptake by DCs and moderately increased the expression of inflammation‐related genes in the nasopharynx lymphoid tissue, the inductive site of common mucosal immune responses 131. Similarly, CHP nanoparticles enhanced efficiency of drug delivery in human hepatocellular carcinoma (HepG2). The findings of *in vitro* experiments suggested that clathrin‐mediated endocytosis and macropinocytosis were involved in the uptake of CHP nanoparticles and the degree of uptake was clearly concentration‐, time‐ and temperature‐dependent. Besides, it was found that nanoparticles were mostly entrapped within the lysosomal apparatus. So, these nanoparticles are a good choice for drug delivery for the treatment of lysosomal storage diseases, cancer and Alzheimer's disease 32.

Pullulan acetate (PA) is known as another important hydrophobized pullulan, which can form self‐aggregated nanoparticles having small particle size, with a narrow size distribution, and a low precipitation rate. *In vitro* study on human nasopharyngeal epidermal carcinoma cell line demonstrated that PA nanoparticles enhanced cellular uptake and mainly intracytoplasmic distribution of epirubicin (EPI) under the same condition when compared with free EPI. As, PA/EPI nanoparticles were taken up by the cells through an endocytic mechanism, hence these particles escape from the endosome/lysosome compartment to enter the cytosol 132.

### Pectin

Pectins typically consist of a complex family of structural polysaccharides present in all plant primary cell walls and contain large quantities of poly‐(d‐galactouronic acid) linked by α‐1,4‐glycosidic bonds. They are random and heterogeneous moieties in terms of chemical composition and molecular mass and can be subdivided as high methoxy (HM), low methoxy (LM) and amidated pectins 133, 134. Pectins remain intact through the stomach and small intestine, while they are degraded by pectinolytic enzymes secreted from bacteria present in the large intestine; therefore, pectin has long been emerged for development of biodegradable carriers for colon‐targeted drug delivery or local treatment of a variety of diseases such as colitis, Crohn's disease and colon carcinomas 22, 135.

Ionotropic gelation and gel coating have been especially suggested to produce pectin‐based drug delivery carriers 134. Pectin nanoparticles are considered as hydrophilic gel nanoparticles which can efficiently encapsulate water‐soluble drugs 136. However, high hydrophilic nature of pectin‐based nanosystems may limit their ability to protect the loaded cargos during gastrointestinal tract 22. This problem can be bypassed by thickening of coating polymer, inactivation of pectinases, application of cross‐linking calcium ions and incorporation of the physical layers that separate drug and pectin 137.

**Figure 10:**
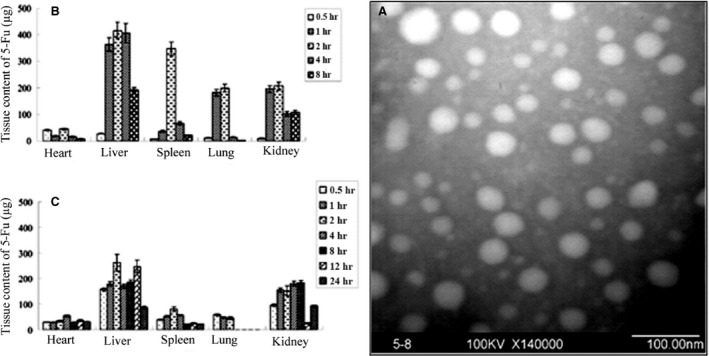
TEM image of pectin‐based nanoparticles (**A**). Content‐time profile of free 5‐FU (**B**) and 5‐FU nanoparticles (**C**) in various tissues at a predetermined time after tail vein i.v. injection at a dose of 5‐FU equivalents to 35 mg/kg (*n* = 3 for each time‐point) (μg) (with permission from authors and Molecular Pharmaceutics) 138.

Recently, citrus pectin and pH‐responsive polymer Eudragit S‐100 have been used to manufacture nanoparticulate carriers for the site‐specific delivery of 5‐fluorouracil (5‐FU) for treatment of colorectal cancer. The results confirmed the ability of nanoparticles to protect the drug loss in the upper parts of GI tract and to deliver 5‐FU when they reach colon owing to the amiable pH of the colonic fluid. Besides, citrus pectin is a specific ligand for galectin‐3 receptors overexpressed in colorectal cancer cells and enhances cellular uptake of the nanoparticles through receptor‐mediated endocytosis 137. Consequently, self‐assembled pectin nanoparticles were examined for delivery of honokiol (HK) in HepG2 expressing high levels of ASGRs and A549 cells. In HepG2 cells, a competitive binding assay in the presence of free galactose showed that uptake mechanism of pectin nanoparticles was significantly dependent on the ASGR‐mediated recognition and endocytosis. In contrast, uptake in A549 cells was not attributed to the ASGR, as the inclusion of free galactose did not show an inhibitory effect on the amount of uptake. Therefore, uptake in A549 cells may be referred to as the non‐specific binding of nanoparticles to the cell surface 136. Another study showed that cellular uptake could be increased when using pectin nanoparticles (Fig. 10A) for delivery of 5‐FU in HepG2 cell line, as compared to free drug. Besides, analysis of tissue distribution after tail vein i.v. injection into healthy mice demonstrated the highest 5‐FU content in the liver, while the distribution of 5‐FU in heart, lung, spleen and kidney was less than that in the liver. Increasing with time, the content of 5‐FU within the body's various tissue was apparently decreased in two free 5‐FU and 5‐FU nanoparticle groups. Eight hours later, no 5‐FU could be found in the free 5‐FU group, but in the 5‐FU nanoparticles group, 24 hrs later the distribution of 5‐FU to various tissues was very low except in the liver and kidney (Fig. 10B and C). It confirmed that the 5‐FU nanoparticles has a prolonged cycle effect compared to that of free 5‐FU^138^.

## Conclusion

Many nanotechnology‐based delivery systems have been designed for the intracellular delivery and subcellular localization of drug/gene. Among these, polysaccharide‐based nanoparticles appear to be a promising carrier in delivering their contents to intracellular targets. The mucoadhesive nature of polysaccharides can provide an opportunity to improve the residence time and to increase binding/uptake properties of the nanoparticles at the absorption site. Moreover, receptor‐targeted polysaccharide nanoparticles can be further applied to enhance the preferential uptake and intracellular accumulation of the pharmaceutical agents. However, a few studies have been conducted to evaluate interaction of the polysaccharide nanoparticles with cell membranes as well as to address their uptake mechanism and their intracellular fate that must be considered.

## Ethical issues

No ethical issues to be promulgated.

## Conflict of interest statement

The authors declare that there are no conflicts of interests.
